# Altered Abundance of Butyrate‐Producing *Lachnospiraceae* by Maternal Diet During Pregnancy Potentially Influences MASLD‐Related Lipid Dysregulation in Male Rat Offspring

**DOI:** 10.1002/mnfr.70153

**Published:** 2025-07-02

**Authors:** Soo‐Min Kim, Sunwha Park, AbuZar Ansari, Gain Lee, Young Min Hur, Jeongshin An, Sang Suk Lee, Young‐Ah You, Young Ju Kim

**Affiliations:** ^1^ Department of Obstetrics and Gynecology College of Medicine Ewha Medical Research Institute Ewha Womans University Mokdong Hospital Seoul Republic of Korea; ^2^ Graduate Program in System Health Science and Engineering Ewha Womans University Seoul Republic of Korea; ^3^ Department of Surgery College of Medicine Institute of Convergence Medicine Research Ewha Womans University Mokdong Hospital Seoul Republic of Korea; ^4^ Anaerobe Laboratory Department of Animal Science and Technology Sunchon National University Jeonnam Suncheon Republic of Korea

**Keywords:** fetal programming, gut microbiome, lipid metabolism, metabolic diseases, short‐chain fatty acids

## Abstract

The maternal diet during pregnancy is an important factor that influences the intrauterine environment during fetal development. However, the relationship among maternal diet, the gut microbiome of offspring, and health outcomes remains unclear. Here, we report that changes in the gut microbiome of offspring after maternal exposure to 50% food restriction and 45% high‐fat diet during pregnancy can affect the risk of metabolic dysfunction‐associated steatotic liver disease (MASLD) in offspring in a sex‐specific manner. Notably, despite no significant difference in body weight, plasma triglyceride and leptin levels were significantly increased in male offspring compared with the controls. The relative abundance of the butyrate‐producing genera of the *Lachnospiraceae* family was dependent on the sex of the offspring and correlated with plasma triglyceride and leptin levels. Interestingly, male offspring in the 50% restricted diet or 45% high‐fat diet groups had reduced butyrate levels compared with the control group and were affected by oxidative damage and hepatic lipogenesis. Our findings suggest that the maternal diet during pregnancy affects the gut microbiota of male offspring in a sex‐specific manner, potentially predisposing them to MASLD later in life through dysregulation of lipid metabolism.

## Graphical abstract

Exposure to undernutrition or overnutrition during the critical period of pregnancy increases susceptibility to metabolic dysfunction‐associated steatotic liver disease (MASLD)–related lipid dysregulation in male rat offspring. The maternal diet during pregnancy altered the relative abundance of specific genera within the *Lachnospiraceae* family and affected butyrate levels in the offspring in a sex‐specific manner. Several key proteins involved in hepatic lipogenesis and GPx activity were also differentially expressed depending on the sex of the offspring. In conclusion, maternal diet during pregnancy influences the composition of butyrate‐producing gut microbiota and butyrate levels in offspring, potentially predisposing them to MASLD later in life in a sex‐specific manner.

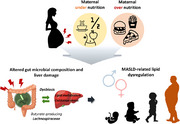

## 1 Introduction

The fetus is programmed during fetal development in the uterus, and the Developmental Origins of Health and Disease theory proposes that environmental factors during early life determine the susceptibility to chronic diseases in adulthood [1, 2, 3, 4]. In particular, animal studies on fetal programming have reported sex‐related differences in pathophysiology, with males being more sensitive to adverse fetal conditions, which may contribute to differences in adult diseases [5, 6, 7]. Additionally, maternal obesity may vertically influence the offspring through dysbiosis of the maternal gut and vaginal microbiota, obesogenic bacteria, and microbial metabolites [8]. However, the effects of maternal undernutrition during pregnancy on the gut microbiome of the offspring and the resulting diseases remain poorly understood.

Newborns are exposed to maternal microbes through vaginal delivery, which is involved in shaping the early gut microbiome and has many implications for the health of the offspring [9, 10, 11]. Microbial diversity is associated with the metabolism by the gut microbiome, and a low richness of the gut microbiota has been consistently implicated as a risk factor for obesity [12, 13]. Short‐chain fatty acids (SCFAs), such as acetate, propionate, and butyrate, which are derived from the gut microbiota through the fermentation of dietary fiber, regulate energy metabolism and the intestinal environment. In addition, butyrate is an important mediator of the gut microbiota and is involved in the regulation of host energy and lipid metabolism through the G protein‐coupled receptor (GPCR) pathway [14, 15]. Several studies have shown that butyrate can alleviate hepatic steatosis associated with metabolic dysfunction‐associated steatotic liver disease (MASLD), and supplements enriched with butyrate‐producing strains such as *Lachnospiraceae* have been reported to exhibit prebiotic effects [16, 17, 18].

The liver plays a crucial role in metabolic homeostasis, being the central organ for gluconeogenesis and lipogenesis [19]. Infants born with intrauterine growth restriction are not only susceptible to several perinatal diseases but are also at high risk for adult conditions such as diabetes, metabolic syndrome, and MASLD [20]. The gut microbiota can ameliorate or exacerbate MASLD through several mechanisms. These include alterations in intestinal permeability, dietary energy intake, de novo lipogenic gene expression, and interactions with the innate immune system [21]. Despite increasing evidence linking alterations in the gut microbiome and metabolic products to metabolic diseases, further research is needed to investigate the effects of maternal diet during pregnancy on the gut microbiome of the offspring in relation to metabolic diseases.

This study aimed to examine whether changes in the gut microbial composition of offspring, resulting from the maternal diet during pregnancy, are associated with blood metabolic parameters and hepatic lipogenesis, thereby contributing to dysregulation of lipid metabolism. To determine the effect of maternal diet on the gut microbiota of the offspring, we investigated sex‐specific changes in the gut microbial composition of the offspring following maternal diet during pregnancy using 16S rRNA sequencing.

## 2 Experimental Section

### 2.1 Experimental Design

All experimental procedures were approved by the Institutional Animal Care and Use Committee of the School of Medicine, Ewha Woman's University (EUM20‐037). Pregnant Sprague‐Dawley rats were purchased from Orient Bio (Orient Bio Inc., Seongnam, Kyunggi‐do, Korea) and acclimated for a week in a controlled environment (12/12 h light/dark cycle, 22°C temperature, and 55% humidity). The compositions of the standard and 45% high‐fat diets are shown in Table S1. Sprague‐Dawley rats were divided into four groups based on their maternal diet during pregnancy and after birth. To induce a 50% food‐restricted diet, the food restriction group dams were fed half the average food intake of control dams from Day 10 of pregnancy. From Day 10 of pregnancy, the dietary regimens included: (1) control group (Control, ad libitum/ad libitum) was fed a standard diet, (2) food restriction group (FR, 50% food restriction/ad libitum) was fed 50% of the consumed by the control group, (3) high‐fat group (HF, 45% high‐fat/ad libitum) was fed a 45% high‐fat diet, and (4) obese group (OB, 45% high‐fat/45% high‐fat) was fed a 45% high‐fat diet both during pregnancy and after birth. After birth, the Control, FR, and HF groups were fed normal standard ad libitum, whereas the OB group was fed 45% high‐fat diet during lactation. Offspring from each group were weighed weekly at the same time until 16 weeks of age, and then sacrificed using anesthesia with a mixture of tiletamine and zolazepam (Zoletil; Virbac Taguig, Philippines) and xylazine (Rompun; Bayer, Germany) administered intramuscularly before exsanguination.

### 2.2 Plasma Metabolic Parameters

Blood samples were collected from rat offspring via cardiac puncture, plasma was obtained by centrifuging whole blood samples at 3000 rpm for 15 min at 4°C, and plasma metabolic parameters were analyzed. Plasma triglyceride concentrations were measured by the enzymatic colorimetric method using Cobas 8000 analyzer (Roche Diagnostics, Mannheim, Germany). Plasma leptin levels were determined with a Leptin ELISA Kit (BioVendor, RD291001200R) using a microplate reader (VersaMax ELISA, Molecular Devices, Sunnyvale, CA, USA) according to the instructions provided by the manufacturer. Free fatty acid (FFA; Abcam, Cambridge, UK), hepatic TG assay (Abcam, Cambridge, UK), and plasma ALT (Abcam, Cambridge, UK) were measured using ELISA kits.

### 2.3 Amplicon Sequence Variant Analysis

Raw Illumina MiSeq data were sequenced and categorized by sample using an index sequence. Paired‐end FASTQ files were generated for each sample. Cutadapt (v3.2) was used to remove the target gene region sequencing adapter and F/R primer sequences, and the forward sequence Read1 and reverse sequence Read2 were cut to 250 and 200 bp, respectively. The DADA2 (v1.18.0) package in the R (v4.0.3) program was used to correct errors in the amplicon sequencing process. Sequences with two or more expected errors were excluded from paired‐end reads. For the preprocessed data, an error model was established for each batch to eliminate noise in each sample. After combining the paired‐end sequences corrected for sequencing errors, the chimeric sequence was eliminated using the consensus method in DADA2 to form amplicon sequence variants (ASVs). ASVs with less than 350 bp were excluded from the pool of ASVs generated using R (v4.0.3). In addition, subsampling was normalized by applying subsampling based on the number of reads of samples with the lowest number of reads among all samples using the QIIME (v1.9) software to compare microbial communities. Each ASV sequence was analyzed using BLAST+ (v2.9.0) in the Reference Database (NCBI 16S Microbial Database) to assign taxonomic information to the most similar organisms. Taxonomic information was not assigned if the query coverage of the top hit matching the database was less than 85% or if the matched area was less than 85% identical. In addition, we used the MAFFT (v7.475) software to perform multiple alignments of ASV sequences and FastTreeMP (v2.1.10) software to generate a phylogenetic tree. Different microbial communities were compared using QIIME and the preceding information on ASV associations and taxonomy. Alpha diversity was confirmed using the rarefaction curve and Chao1 values, and the species diversity and equality of microbial communities in the samples were checked using Shannon and Inverse Simpson indices. Based on the weighted and unweighted UniFrac distance calculations, we obtained beta diversity between the samples. We then used principal coordinates analysis (PCoA) and the unweighted pair‐group method with an arithmetic mean tree to visualize their relationships.

### 2.4 Metabolites

Volatile fatty acid concentrations were analyzed by high‐performance liquid chromatography (Agilent Technologies 1200 series, Tokyo, Japan) with a UV detector set at 210 and 220 nm. Samples were eluted isocratically with 0.0085 N H_2_SO_4_ at a 0.6 mL/min flow rate and 35°C column temperature.

### 2.5 Western Blotting

Liver tissues were homogenized and lysed in radioimmunoprecipitation assay buffer (Biosesang, Seongnam, Korea) mixed with a protease inhibitor cocktail (Roche Diagnostics GmbH, Mannheim, Germany). The supernatants were then centrifuged at 12 000 rpm for 20 min at 4°C, and protein concentrations in supernatants were measured using a BCA protein assay kit (Thermo Fisher Scientific, Rockford, IL, USA). Equal amounts of a sample containing 40 µg protein were separated using SDS‐PAGE and transferred onto a polyvinylidene fluoride (PVDF) membrane (Cytiva, Freiburg im Breisgau, Germany) at 80 V for 2 h. The membranes were blocked for 2 h with 5% (w/v) BSA in Tris‐buffered saline containing 0.01% Tween‐20 (TBST). Then, they were incubated with anti‐fatty acid synthase (FASN) (1:1000, Santa Cruz Biotechnology, Texas, USA) antibodies in TBST buffer containing 1% BSA for 3 days at 4°C. Additionally, they were incubated overnight at 4°C with microsomal triglyceride transfer protein (MTP) (1:1000, Santa Cruz Biotechnology, Texas, USA), and beta‐actin (β‐actin, 1:1000, Santa Cruz Biotechnology, Texas, USA) antibodies. After washing with TBST, the membranes were incubated with mouse IgGk BP‐horseradish peroxidase (Santa Cruz Biotechnology, Texas, USA) and mouse anti‐rabbit IgG‐horseradish peroxidase (Santa Cruz Biotechnology, Texas, USA) secondary antibodies for 1 h at 25°C. Finally, the membranes were washed with TBST, and protein bands on the membranes were developed and detected using Western Blotting Luminol Reagent (Santa Cruz Biotechnology). β‐Actin normalized band intensity and relative band intensity were quantified using the ImageJ software.

### 2.6 Statistical Analysis

Data are presented as the mean ± standard error of the mean. Statistical analysis was performed using SPSS software (ver. 18.0.0; Chicago, IL, USA). One‐way analysis of variance (ANOVA) followed by Dunnett's post‐hoc test was used for normally distributed data, whereas the Kruskal–Wallis test followed by the Mann–Whitney post‐hoc test was used for non‐normally distributed data. Statistical analysis for interactions with group and sex as factors was performed using two‐way ANOVA, followed by Tukey's post‐hoc test. Statistical significance was set at *p <* 0.05. The box plot represents the interquartile range (IQR; 25–75th percentiles), the horizontal line within the box indicates the median, and the whiskers extend to 1.5 times the IQR.

## 3 Results

### 3.1 Characteristics of 16‐Week‐Old Rat Offspring

**FIGURE 1:**
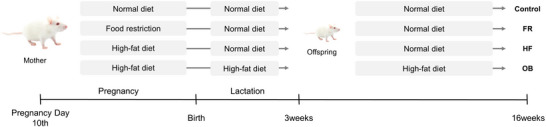
Experimental design. Sprague‐Dawley rats are divided into four groups based on the maternal diet during pregnancy and lactation: (i) control group (Control, ad libitum/ad libitum), (ii) food restriction group (FR, 50% food restriction/ad libitum), (iii) high‐fat group (HF, 45% high‐fat/ad libitum), and (iv) obese group (OB, 45% high‐fat/45% high‐fat). After lactation, offspring in the Control, FR, and HF groups are fed a standard diet until 16 weeks of age, whereas offspring in the OB group are fed a 45% high‐fat diet. FR indicates food restriction; HF, high‐fat diet; OB, obese.

To assess alterations in the gut microbiome of mothers and offspring according to prenatal diet, we divided rats into four groups based on the maternal diet from Day 10 of pregnancy: ad libitum/ad libitum (Control), 50% food restriction*/*ad libitum (FR), 45% high‐fat/ad libitum (HF), and 45% high‐fat/45% high‐fat (OB) (Figure 1). To confirm the effects of maternal diet during pregnancy on offspring, Control, FR, and HF offspring were fed a normal diet at 3 weeks of age, at the end of lactation. The offspring of the OB group were fed a 45% high‐fat diet until 16 weeks of age and were compared with the other groups as a control for obesity.

**FIGURE 2:**
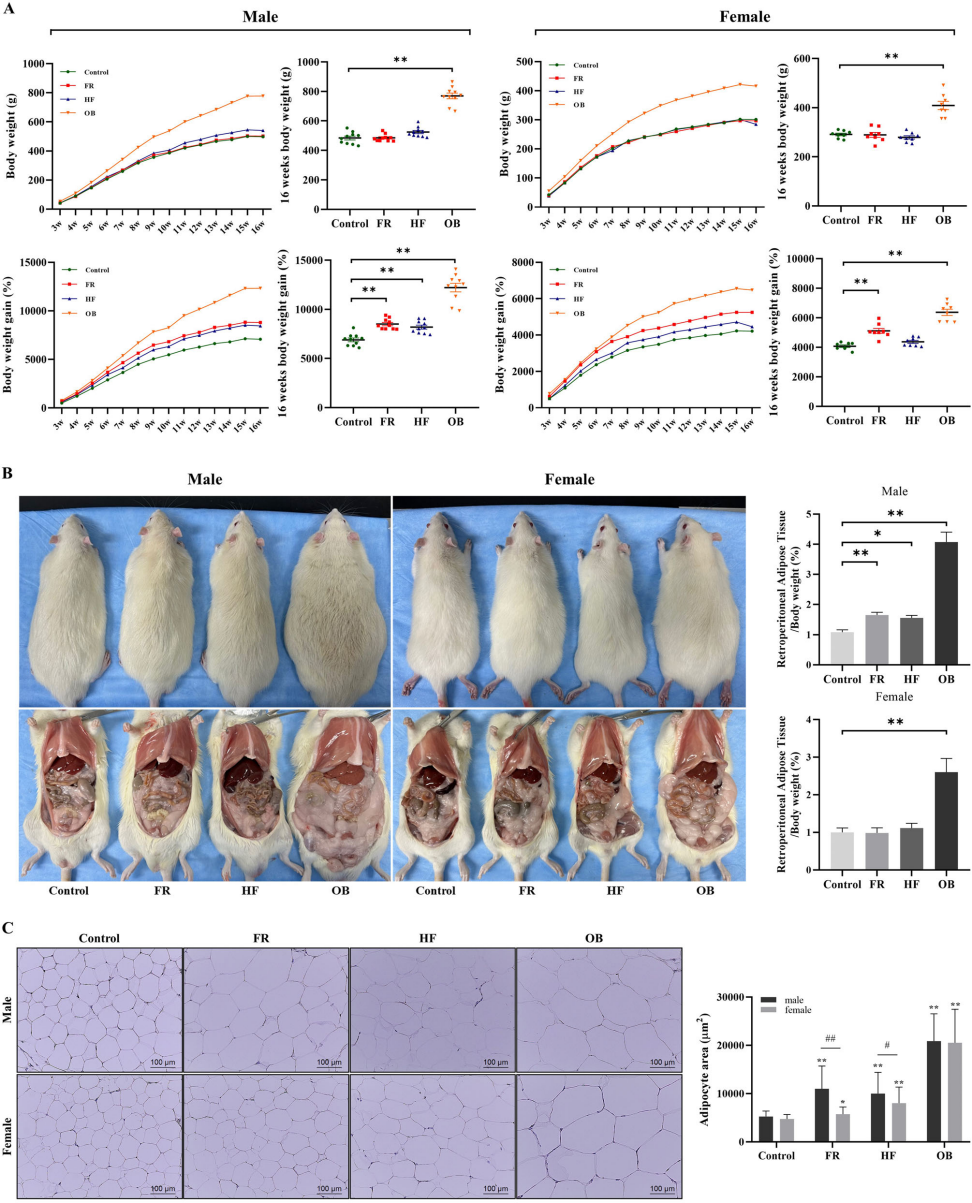
Effects of maternal diet during pregnancy on body weight and adipose tissue growth in 16‐week‐old rat offspring. (A) Comparison of body weight and body weight gain in rat offspring. Effect of maternal 50% food restriction and 45% high‐fat diet during pregnancy on catch‐up growth at 16 weeks of age in offspring (n = 10 males and n = 8 females in each group). Differences between each group and control group are determined using ANOVA, followed by the Kruskal–Wallis test and Mann–Whitney post‐hoc test. Data are presented as mean ± standard error of the mean (SEM). (B) Representative photographs and comparison of adipose tissue weight in rat offspring. (C) Adipose tissue of rat offspring stained with H&E in the Control, HF, and OB groups. Adipocyte area is determined using the ImageJ software. Magnification × 200; scale bar 100 µm; * *p <* 0.05 versus Control, ** *p <* 0.001 versus Control, # *p <* 0.05 male versus female, ## *p <* 0.001 male versus female.

Newborns were weighed at birth and every week afterwards, and the offspring were sacrificed at 16 weeks of age. Although the birth weights of offspring in the maternal FR and HF groups were significantly lower than that of the control group offspring (Figure S1), the body weights of male and female offspring in both groups at 16 weeks of age were similar to that of the control group (Figure 2A). However, weight gain, calculated as the ratio of the body weight of the offspring at 16 weeks of age to their birth weight, showed a significant increase in male and female FR offspring and male HF offspring compared with the control group (*p <* 0.001). In addition, weight gain was consistently higher in the FR group than in the HF group. Male and female OB offspring had significantly higher body weight and ratio of body weight gain to birth weight than those of the control group (*p <* 0.001). Male offspring had higher body weight and weight gain than those of females, and the sex‐interaction effects were significant for body weight (*F* = 24.986, *p <* 0.001) and weight gain (*F* = 19.453, *p <* 0.001). There were significant differences in retroperitoneal white adipose tissue (rWAT) between male and female offspring (Figure 2B). The weight of rWAT was significantly increased in all male groups and only the female OB group compared with that of the control. Adipocytes were larger in both male and female offspring of the FR, HF, and OB groups than those in the control group, based on H&E staining (Figure 2C). Specifically, the adipocyte area in the FR and HF male offspring was significantly larger than that in the FR and HF female offspring.

### 3.2 Plasma Lipid Profiles of 16‐Week‐Old Offspring

**FIGURE 3:**
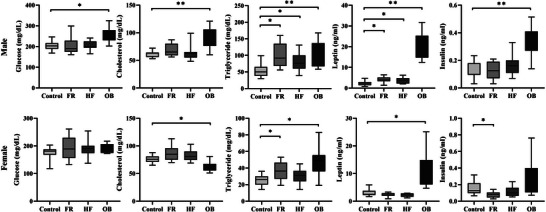
Plasma endocrine and metabolic markers in rat offspring. Comparison of plasma metabolic parameters between male and female rat offspring (*n* = 10 males and *n* = 8 females in each group). Blood samples from rat offspring collected via cardiac puncture are used to assess plasma levels of metabolic markers. Metabolic parameters are measured using ELISA following the instructions provided by the manufacturer. Box plots show the interquartile range (25–75th percentile), with whiskers extending to the minimum and maximum values. Differences between each group and the control group are determined using ANOVA, followed by the Kruskal–Wallis test and Mann–Whitney post‐hoc test. The interaction effects with group and sex are analyzed using two‐way ANOVA, followed by Tukey's post‐hoc test. * *p <* 0.05 versus Control, ** *p <* 0.001 versus Control.

We analyzed the plasma glucose and lipid profiles of rat offspring (Figure 3). Glucose and total cholesterol levels were significantly increased in male OB offspring (*p <* 0.05 and *p <* 0.001, respectively), but total cholesterol levels were significantly decreased in female OB offspring (*p <* 0.05). Triglyceride levels were significantly higher in both males and females than in the control group. In males, triglyceride levels were significantly increased in the FR, HF, and OB groups (*p <* 0.05), and in female offspring of the FR and OB groups (*p <* 0.05). Specifically, sex differences were observed in leptin levels, with significant increases in FR, HF, and OB male offspring. Leptin levels according to group were significantly different with interaction effect by sex (*F* = 8.698, *p <* 0.001), and in all groups, except Control, leptin levels were higher in males than in females. Insulin levels increased in male offspring in the OB group but decreased in female offspring in the FR group.

### 3.3 Gut Microbiome Analysis

To compare the gut microbial composition, ASV analysis after 16S rRNA sequencing was conducted on the colon tissues from mothers and rat offspring. First, although not significant, the alpha diversity showed that male offspring of the FR, HF, and OB groups (Figure 4A) had lower species richness (Chao1 and ASVs) and evenness (Shannon and Gini‐Simpson index) than those of the control group. However, female offspring of the FR and HF groups showed a trend toward a decrease in evenness, but no difference in richness compared to the control group (Figure 4A). Maternal gut microbiota richness was also marginally decreased in the FR, HF, and OB groups compared with that in the control group, showing similarities to male offspring (Figure S2). To compare the composition of the gut microbial communities, we performed PCoA using unweighted/weighted UniFrac values in taxonomic distribution configurations among each group of offspring (Figure 4B). Unweighted/weighted UniFrac analysis showed a significant difference among the four groups and between male and female offspring (*p <* 0.001). In terms of the relative abundance of the top 20 most dominant genera in male offspring, the dominant genus was *Lactobacillus*, followed by *Akkermansia*, *Bacteroides*, and *Lomboutsia* (Figure 4C). However, the most prominent genus in female offspring was *Akkermansia*, followed by *Lomboutsia*, *Bacteroides*, and *Parabacteroides*. The heat map analysis presented the relative abundance of genera in mothers (Figure S2) and male and female offspring, and the differences in the dominant bacterial genera among different groups (Figure 4D). The sample groups are displayed above the heat map in green (Control), orange (FR), blue (HF), and yellow (OB). In male offspring, the Control, HF, and OB groups were clustered. In particular, *Lachnospira* showed different intensities in the FR, HF, and OB groups compared with the control.

FIGURE 4Comparison of alpha diversity and relative abundance of the gut microbiota in rat offspring. (A) Alpha diversity richness (Chao1 and ASVs) and evenness (Shannon and Gini‐Simpson indices) of the gut microbiota in rat offspring. Box plots show the interquartile range (25–75th percentile), with whiskers extending to the minimum and maximum values. (B) Beta diversity visualized with PCoA using unweighted/weighted UniFrac distance within offspring groups. (C) Top 20 most dominant genera in male and female offspring. (D) Heat map of the relative abundance at the genus level in mothers and male and female offspring. Each group consists of three males and four females. C indicates class; F, family; G, genus; O, order; P, phylum; S, species.

**Figure:**
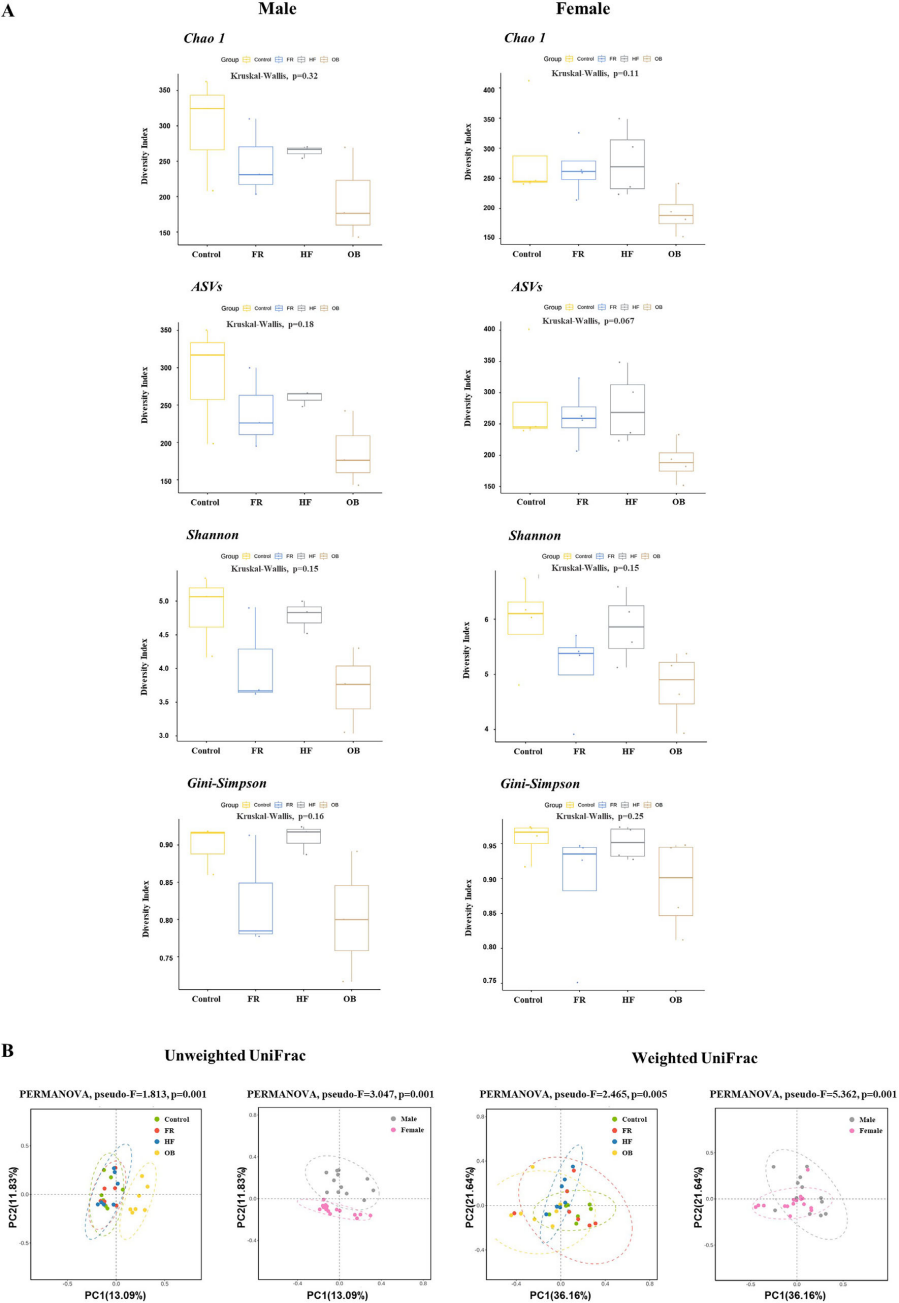

**Figure:**
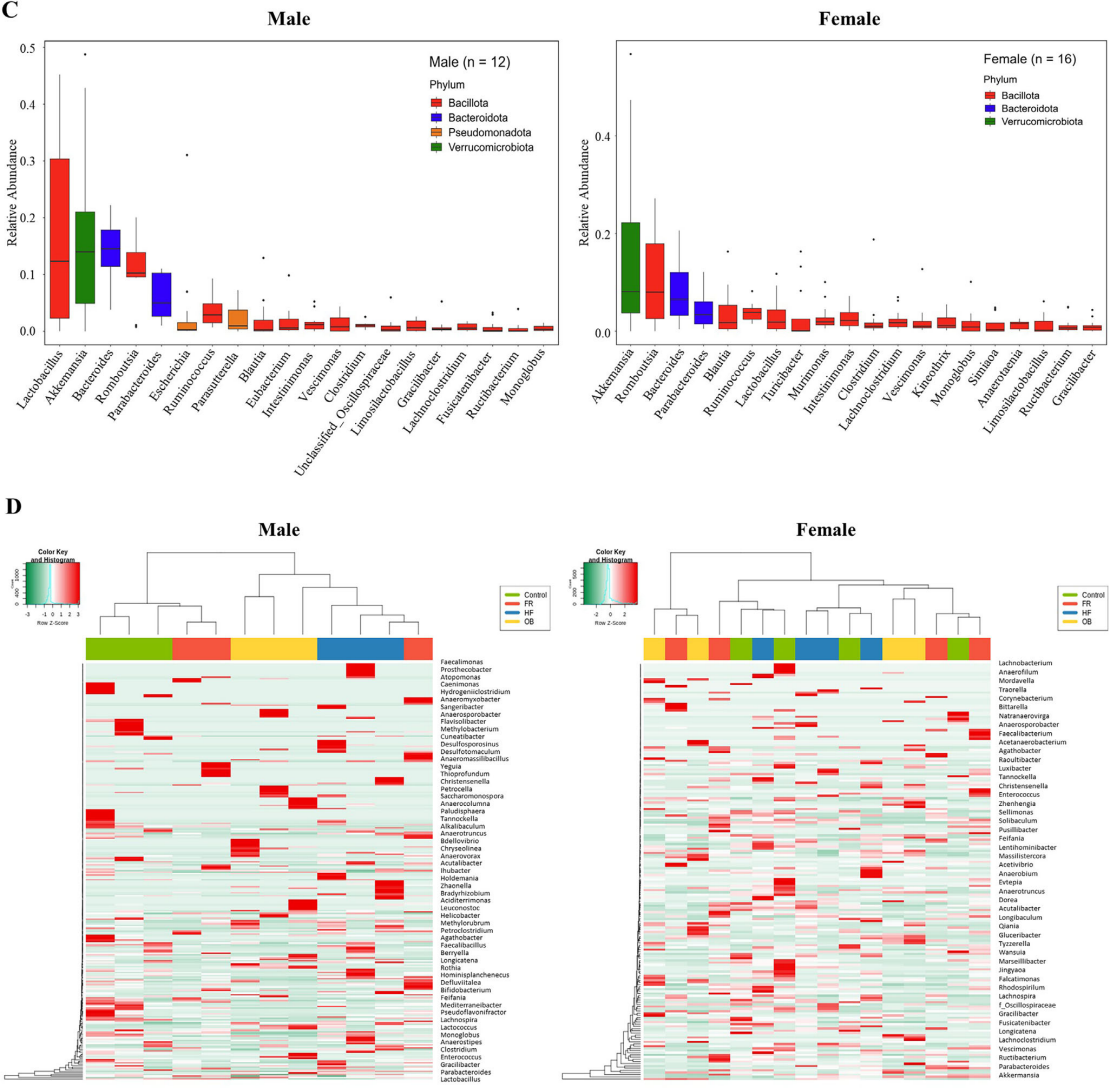

The linear discriminant analysis effect size (LEfSe) cladogram showed a difference in the composition of the dominant bacteria between the Control and other groups (Figure 5). Significantly lower relative abundance of a few genera belonging to butyrate‐producing *Lachnospiraceae* was observed in mothers and male rat offspring of the FR, HF, and OB groups than in the Control group. Among these, relative abundance of the genus *Anaerostipes* decreased in the FR and OB groups (*p <* 0.001), whereas that of *Lachnospira* decreased in the HF and OB groups. Interestingly, these alterations were observed in both mothers and male offspring (*p <* 0.001). However, in female offspring of the FR and HF groups, the microbiota composition showed fewer differences in the dominant bacteria compared to the Control group (Figure S3).

FIGURE 5Taxonomy cladogram generated using linear discriminant analysis effect size (LEfSe) analysis. Classification diagram produced using LEfSe analysis in mothers and male offspring. This cladogram shows different levels of classification arranged in concentric circles. The outer circles represent phyla, whereas the inner circles represent species. The diagram highlights specific bacterial taxa that are distinctive in the FR(A), HF(B), and OB(C) compared with the Control group, and these distinctions are identified using LEfSe. Box plots show the interquartile range (25th–75th percentile), and whiskers represent data points within 1.5 × IQR from the quartiles. Each group consists of three males and four females. Histogram displays the scores of linear discriminant analysis (LDA > 2; Figure S4).

**Figure:**
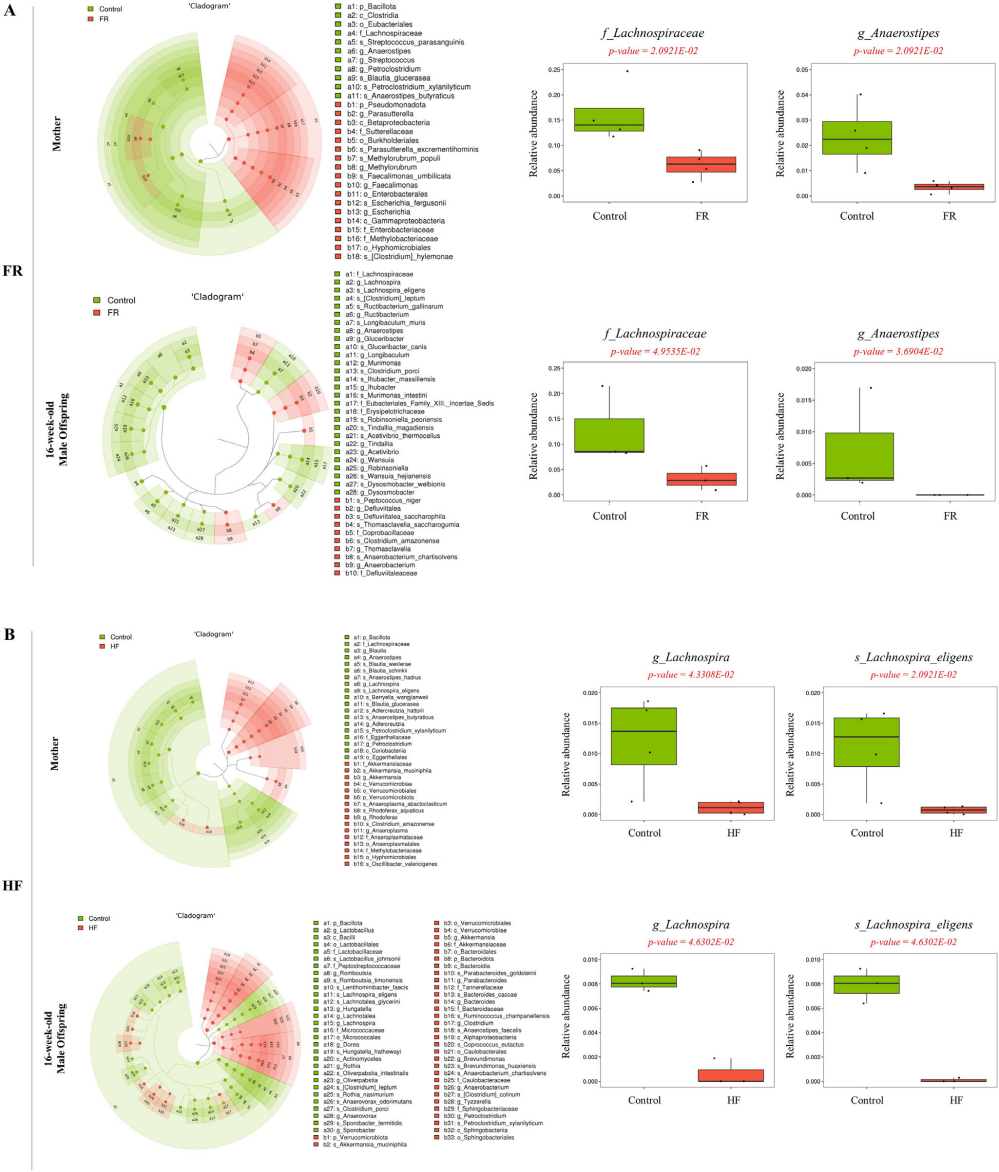

**Figure:**
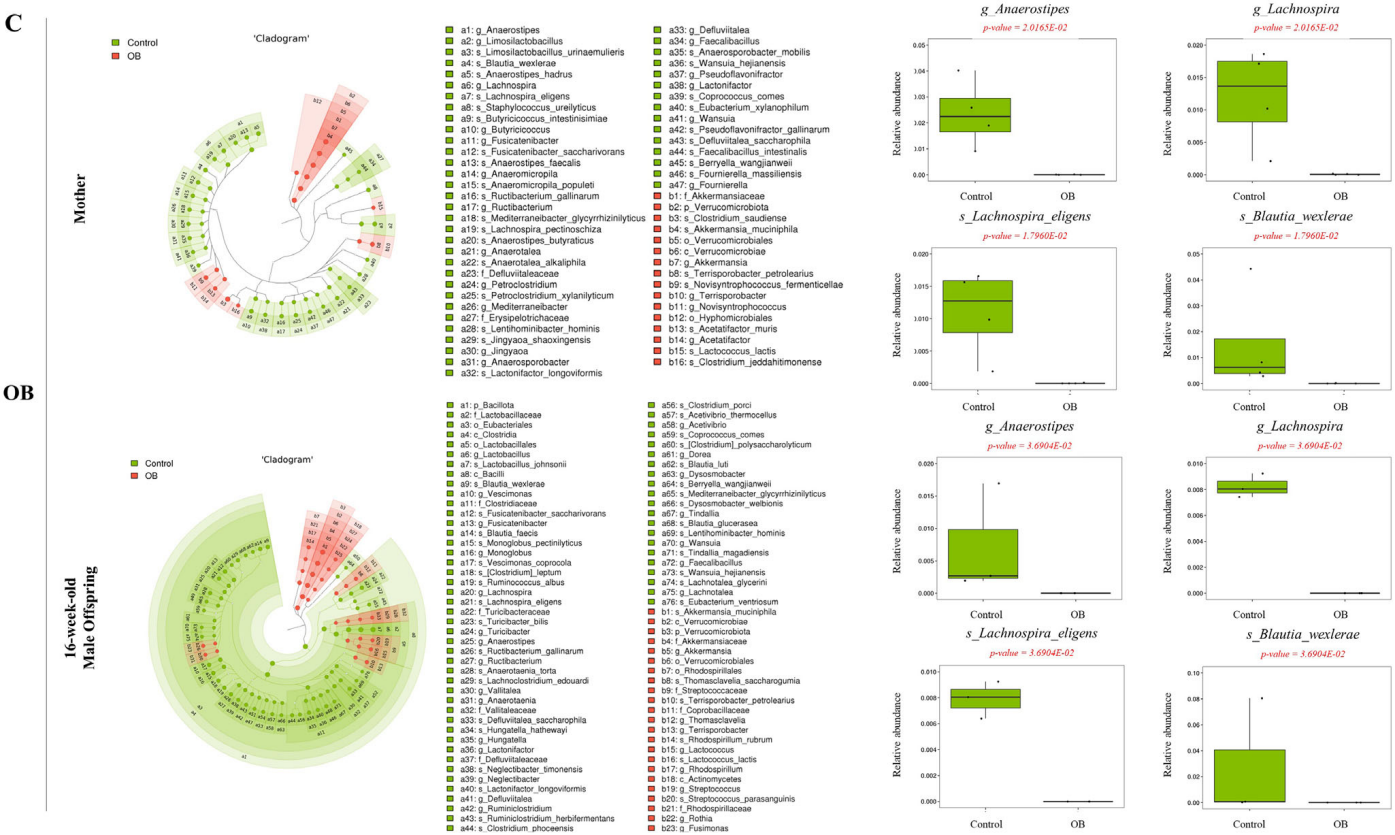

**FIGURE 6:**
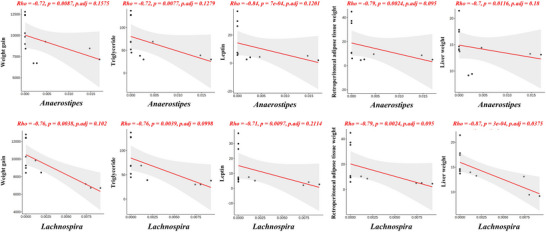
Correlation between bacterial genera belonging to *Lachnospiraceae* and metabolic parameters. Correlation analysis between metabolic parameters and *Lachnospiraceae* genus levels in male offspring (*n* = 12). Following Spearman's correlation analysis, the Benjamin‐Hochberg method is used to control the false discovery rate.

We examined this correlation to evaluate the effect of the gut microbiome on metabolic parameters, including plasma triglyceride and leptin levels (Figure 6). Specifically, weight gain, triglyceride, leptin, retroperitoneal adipose tissue, and liver weight were negatively associated with *Anaerostipes* and *Lachnospira*, which belong to the *Lachnospiraceae* family.

### 3.4 Analysis of SCFAs

**FIGURE 7:**
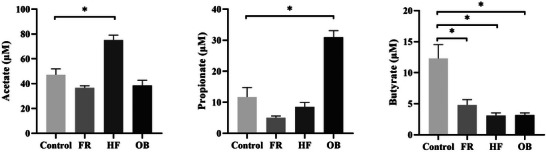
Comparison of plasma levels of short‐chain fatty acids (SCFAs) in male offspring. Comparison of SCFAs measured using liquid chromatography‐mass spectrometry in the Control, FR, HF, and OB groups. Five males are included in each group. Data are presented as mean ± SEM. Differences between each group and control group are determined using one‐way ANOVA, followed by Dunnett's post‐hoc test. * *p <* 0.05 versus Control, ** *p <* 0.001 versus Control.

To determine the effect of reduced SCFA‐producing gut microbiota, we measured SCFAs in male offspring (Figure 7). Plasma acetate concentration increased in the HF group, and propionate concentration increased in the OB group compared with that in the Control group. Notably, plasma butyrate concentration decreased in all groups compared with the Control group (*p <* 0.05).

### 3.5 Hepatic Metabolism

**FIGURE 8:**
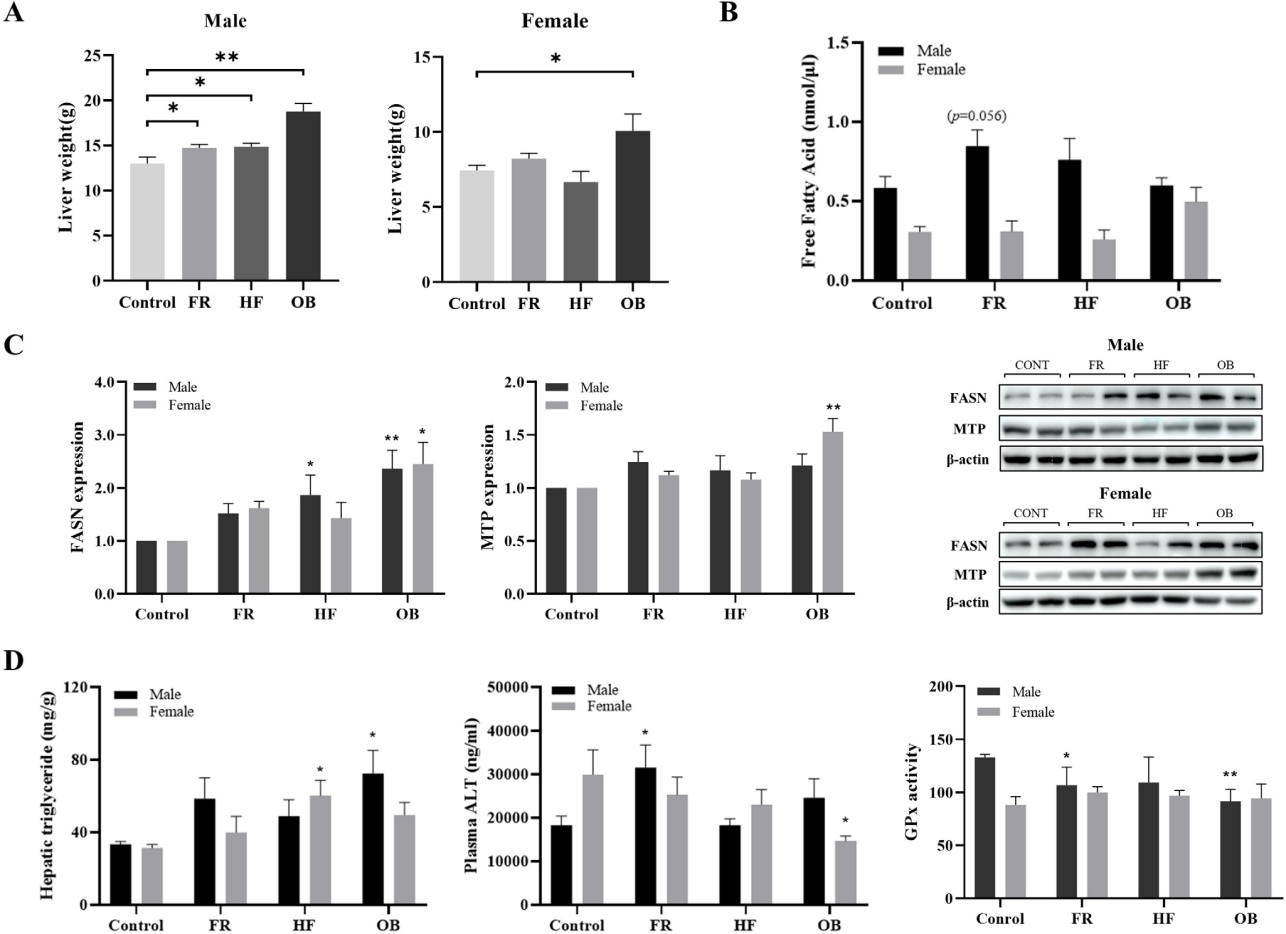
Parameters of hepatic metabolic dysregulation involving lipid metabolism and oxidative damage. (A) Comparison of liver weight of the offspring (*n* = 10 males and *n* = 8 females in each group). (B) The levels of FFA in plasma were determined by ELISA. (C) Protein expression levels linked to lipid metabolism (FASN and MTP) are measured in the liver using western blotting. A graphical representation of the intensity ratios is obtained using the ImageJ software. (D) Hepatic triglyceride, plasma ALT, and GPx activity were measured by ELISA and colorimetric assay. Data are presented as mean ± SEM. Differences between each group and the control group were determined using one‐way ANOVA, followed by Dunnett's post‐hoc test. * *p <* 0.05 versus Control, ** *p <* 0.001 versus Control.

The liver weights of the male FR, HF, and OB groups, as well as the female OB group, were significantly higher than those of the Control group (*p <* 0.05) (Figure 8A). Although not statistically significant, plasma FFA levels showed a marginal increase in the male FR group (Figure 8B). Similarly, the protein expression levels of FASN and MTP tended to increase in this group (Figure 8C). In addition, FASN levels were significantly higher in male HF and OB offspring, as well as in female OB offspring, compared to the Control group (*p <* 0.05). However, MTP expression was significantly increased only in female OB offspring (*p <* 0.001). Hepatic triglyceride (TG) levels also tended to increase in the male FR group, and plasma alanine aminotransferase (ALT), an indicator of liver damage, was significantly elevated in the male FR group (Figure 8D). We also assessed hepatic glutathione peroxidase (GPx) activity and lipid metabolism in offspring and observed sex‐related differences (Figure 8D). Compared with the Control group, liver GPx activity was decreased in male FR and OB offspring.

## 4 Discussion

This study demonstrates that maternal dietary patterns during pregnancy influence the gut microbiota composition of offspring, particularly affecting the abundance of butyrate‐producing *Lachnospiraceae* in a sex‐specific manner. These alterations are associated with dysregulated lipid metabolism and early markers of hepatic steatosis in male offspring, potentially increasing their susceptibility to MASLD later in life. Notably, both maternal undernutrition (50% food restriction) and overnutrition (a 45% high‐fat diet) during pregnancy resulted in similar metabolic perturbations in male offspring, with undernutrition producing more pronounced effects on plasma ALT levels and oxidative stress markers, despite the offspring being maintained on a standard diet after birth.

The fetal origins of adult disease hypothesis postulates that environmental factors during early development have a significant impact on an individual's susceptibility to diseases in adulthood [22]. Large birth registries and human cohorts in which pregnant women and their offspring experienced extreme malnutrition in the form of famine initially supported this theory [3, 22, 23, 24, 25]. Our previous studies have shown that maternal malnutrition alters blood lipid and carbohydrate metabolite profiles, as well as one‐carbon and lipid metabolism, by altering gene expression in the liver [26, 27, 28, 29]. In the present study, we confirmed that male offspring of mothers exposed to undernutrition or overnutrition during pregnancy, in particular, had different plasma triglyceride and leptin levels.

It has been postulated that the infant gut microbiome is affected by the vertical transfer of the maternal gut microbiome following vaginal delivery and breastfeeding, and that the gut microbial composition differs between males and females through the interaction of sex hormones with the gut microbiota [30, 31]. In addition, low alpha diversity in the gut microbiota has been linked to metabolic diseases [32, 33]. Early gut microbial dysbiosis is associated with higher levels of potentially pathogenic microbes, and low‐grade inflammation interacts with the gut microbiome, leading to obesity in children and adults [34]. In this study, maternal diet during pregnancy influenced the gut microbial composition in male offspring. Alpha diversity was reduced in both mothers and male offspring in the FR and OB groups compared with that in the control group. This suggests that the potential influence of maternal diet on alpha diversity in the offspring is sex specific.

Gut microbial communities influenced by the host diet can cause metabolic dysfunction by producing metabolites, such as SCFAs, and are also associated with clinical blood markers [35, 36]. *Lachnospiraceae* has been known to play a critical role in producing butyrate. Several studies have shown that SCFAs produced by *Lachnospiraceae* improve inflammation by providing energy to the intestine and positively affect various metabolic and intestinal diseases [37, 38, 39, 40, 41]. Additionally, reduced SCFA levels may result in increased intestinal permeability, which can be a significant pathogenic factor in MASLD [35, 42, 43, 44]. In this study, the gut microbiota belonging to the *Lachnospiraceae* family altered in male offspring as well as in mothers. Consistent with the decrease in butyrate‐producing gut bacteria, we confirmed that plasma butyrate levels were reduced. Additionally, a correlation was found between the abundance of a specific genus of *Lachnospiraceae* and concentrations of plasma triglycerides and leptin. These results suggest that some genera of the maternal gut microbiome established during pregnancy can be transmitted to offspring and influence SCFA production and blood lipid parameters.

The liver is responsible for many essential metabolism‐related biological functions, and has been implicated in the development of metabolic syndromes. In particular, abnormal accumulation of triglycerides in the liver is the main feature of fatty liver disease. Additionally, FASN is associated with lipogenesis and MTP is essential for VLDL secretion. Upregulation of these genes, which are associated with hepatic lipogenesis, is specifically linked to the development of hepatic steatosis and MASLD [45, 46]. Hepatic steatosis leads to elevated FFA and increased hepatic triglyceride levels. This lipid accumulation in the liver causes lipotoxicity and oxidative stress, which can result in hepatic dysfunction and may progress to MASLD [47]. GPx is an antioxidant enzyme that protects against oxidative stress, and it is known that oxidative stress is involved in the pathogenesis of MASLD. A study in adolescents found a significant correlation between GPx levels and MASLD [48], and an animal study confirmed that the consumption of beneficial bacteria improved GPx activity in obese rats fed a high‐fat diet [49]. In this study, plasma FFA and hepatic TG tended to increase, while plasma ALT levels and GPx activity were significantly decreased in the male FR group compared to controls. These results suggest that maternal diet during pregnancy may alter the butyrate‐producing gut microbiome in a sex‐dependent manner, leading to lipid metabolism dysregulation and liver injury that may contribute to the early development of MASLD.

This study has limitations. It did not confirm the influence of the gut microbiome on adult offspring according to the maternal diet during pregnancy. Therefore, studies should be conducted to identify changes in the gut microbiome according to maternal diet during pregnancy across the life cycle, from birth to adulthood, and how these changes affect metabolic diseases, including obesity. However, this study is significant because it confirms that maternal diet during pregnancy may increase the risk of metabolic diseases in offspring by comparing the gut microbial composition of young adult offspring according to sex. Our results suggest that maternal undernutrition during pregnancy may have more adverse effects than that of overnutrition. Further research is required to understand the mechanisms underlying the development of adult metabolic disease‐associated gut microbiome influenced by maternal nutrition in early life.

In conclusion, the gut microbial composition and metabolites of young adult offspring are significantly influenced by maternal diet during pregnancy in a sex‐specific manner. Butyrate deficiency is associated with elevated plasma leptin and FFA levels, which, in turn, promote hepatic lipogenesis and impair antioxidant defenses, ultimately leading to hepatic triglyceride accumulation and systemic dysregulation of lipid metabolism. These changes may also affect susceptibility to MASLD later in life. In particular, maternal malnutrition may serve as a high‐risk factor for MASLD associated with lipid metabolism dysregulation in the offspring, even when they are fed a standard diet after birth. Our data suggest that alterations in the gut microbiome due to maternal diet could serve as potential early markers for predicting MASLD risk, particularly in male offspring

## Conflicts of Interest

The authors declare no conflicts of interest.

## Supporting information




**Supporting Information file 1**: mnfr70153‐sup‐0001‐SupMat.pdf

## Data Availability

All data generated or analyzed during this study are included in this published article and its supporting information files or are available from the corresponding author upon reasonable request.
