# Supplementary material for: Altered Abundance of Butyrate‐Producing Lachnospiraceae by Maternal Diet During Pregnancy Potentially Influences MASLD‐Related Lipid Dysregulation in Male Rat Offspring
Source: Mol Nutr Food Res. 2025 Jul 2;69(19):e70153. doi: 10.1002/mnfr.70153 (PMC12490193; doi:10.1002/mnfr.70153)
Supplement: Supplementary file 1 — Supporting Information file 1: mnfr70153‐sup‐0001‐SupMat.pdf [file MNFR-69-e70153-s001.pdf]

Altered abundance of butyrate-producing *Lachnospiraceae* due to maternal diet during pregnancy potentially affects hepatic metabolism in male rat offspring

Soo-Min Kim[1,2], Sunwha Park[1], AbuZar Ansari[1], Gain Lee[1,2], Young Min Hur[1], Jeongshin An[3], Sang Suk Lee[4], Young-Ah You[1]* and Young Ju Kim[1,2]*

[1]Department of Obstetrics and Gynecology, College of Medicine, Ewha Medical Research Institute, Ewha Womans University Mokdong Hospital, Seoul, Republic of Korea

[2]Graduate Program in System Health Science and Engineering, Ewha Womans University, Seoul, Republic of Korea

[3]Department of Surgery, College of Medicine, Institute of Convergence Medicine Research, Ewha Womans University Mokdong Hospital, Seoul, Republic of Korea

[4]Anaerobe Laboratory, Department of Animal Science and Technology, Sunchon National University, 413 Jungangno, Jeonnam, Suncheon 57922, Republic of Korea

***Correspondence: Young-Ju Kim and Young-Ah You**

kkyj@ewha.ac.kr (Young Ju Kim) and yayou@ewha.ac.kr (Young-Ah You)

Keywords**:** fetal programming, gut microbiome, lipid metabolism, metabolic diseases, short-chain fatty acids

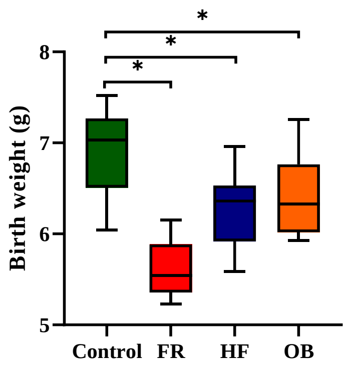

Figure S1. The weight of the offspring at birth.

Birth weight measured in Control, FR, HF, and OB groups immediately after birth. * $p < 0.05$ versus Control, ** $p < 0.001$ versus Control.

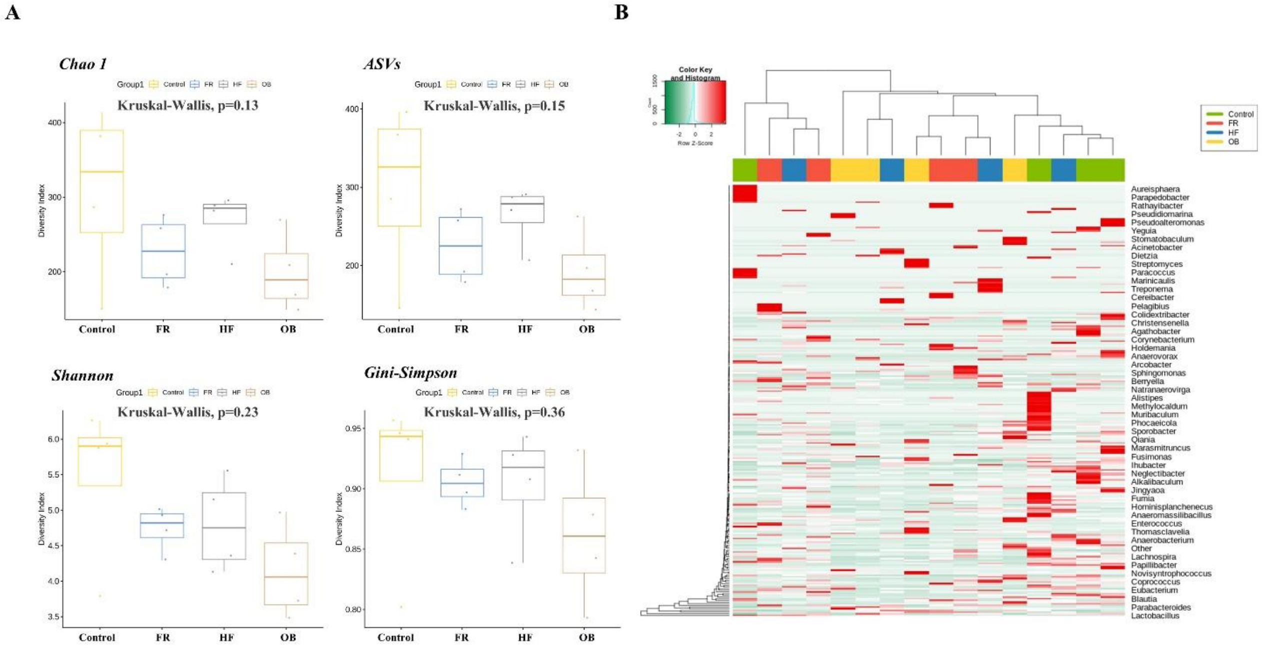

Figure S2. Comparison of gut microbiota alpha diversity and relative abundance in maternal groups.

(A) The alpha diversity richness (Chao 1and ASVs) and evenness (Shannon and Gini-Simpson index) of the gut microbiota in FR, HF, and OB maternal groups. **(B)** Heatmap of the relative abundance at the genus level in maternal groups. (P = phylum; C = class; O = order; F = family; G = genus; S = species).

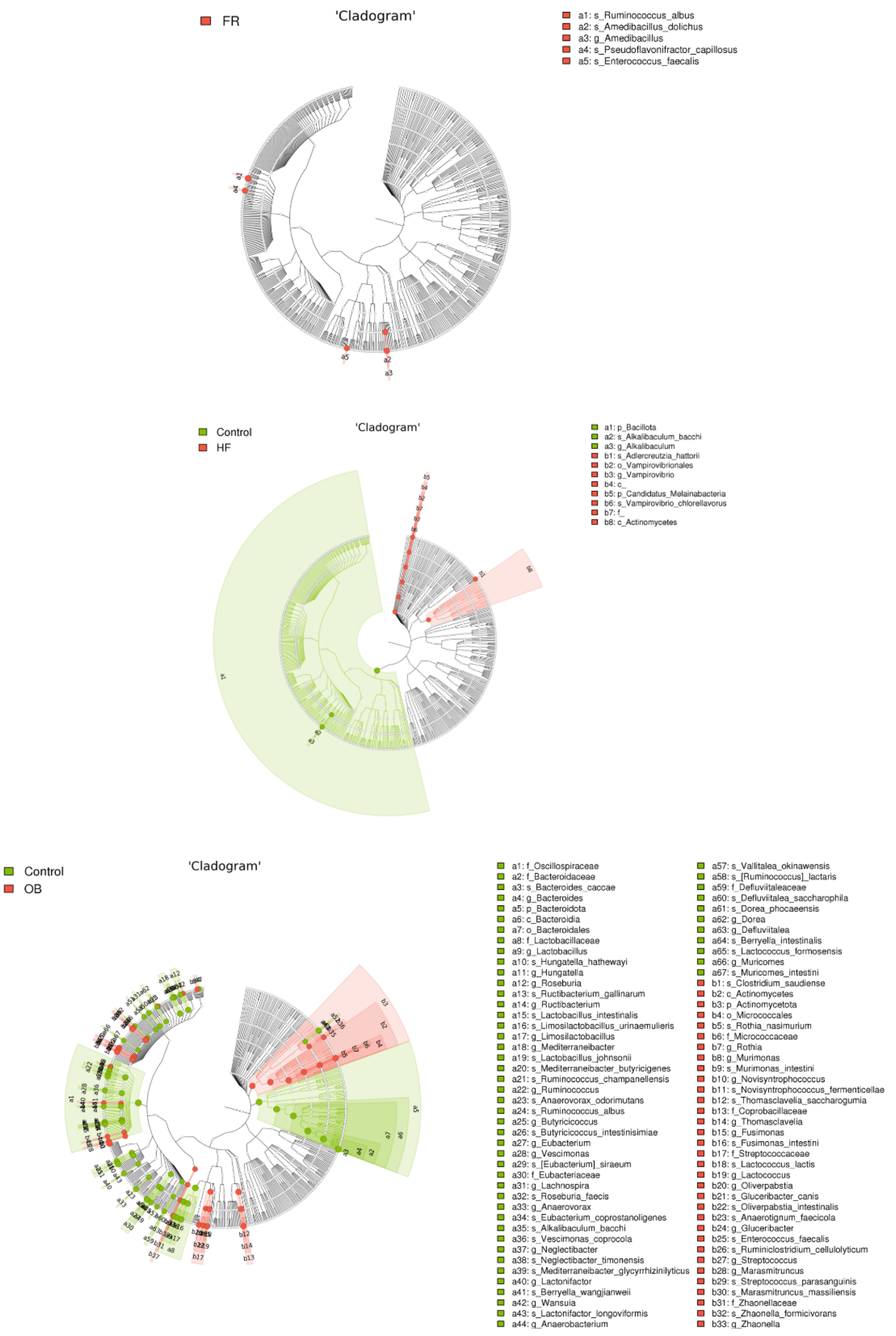

Figure S3. Taxonomy cladogram generated using linear discriminant analysis effect size (LEfSe) analysis.

Classification diagram produced using LEfSe analysis in 16-week-old female offspring group. This cladogram shows different levels of classification arranged in concentric circles. The outer circles represent phyla, whereas the inner circles represent species. The diagram highlights specific bacterial taxa that are distinctive in the three different groups (FR, HF, and OB) when compared with the control groups, and these distinctions were identified using LEfSe.

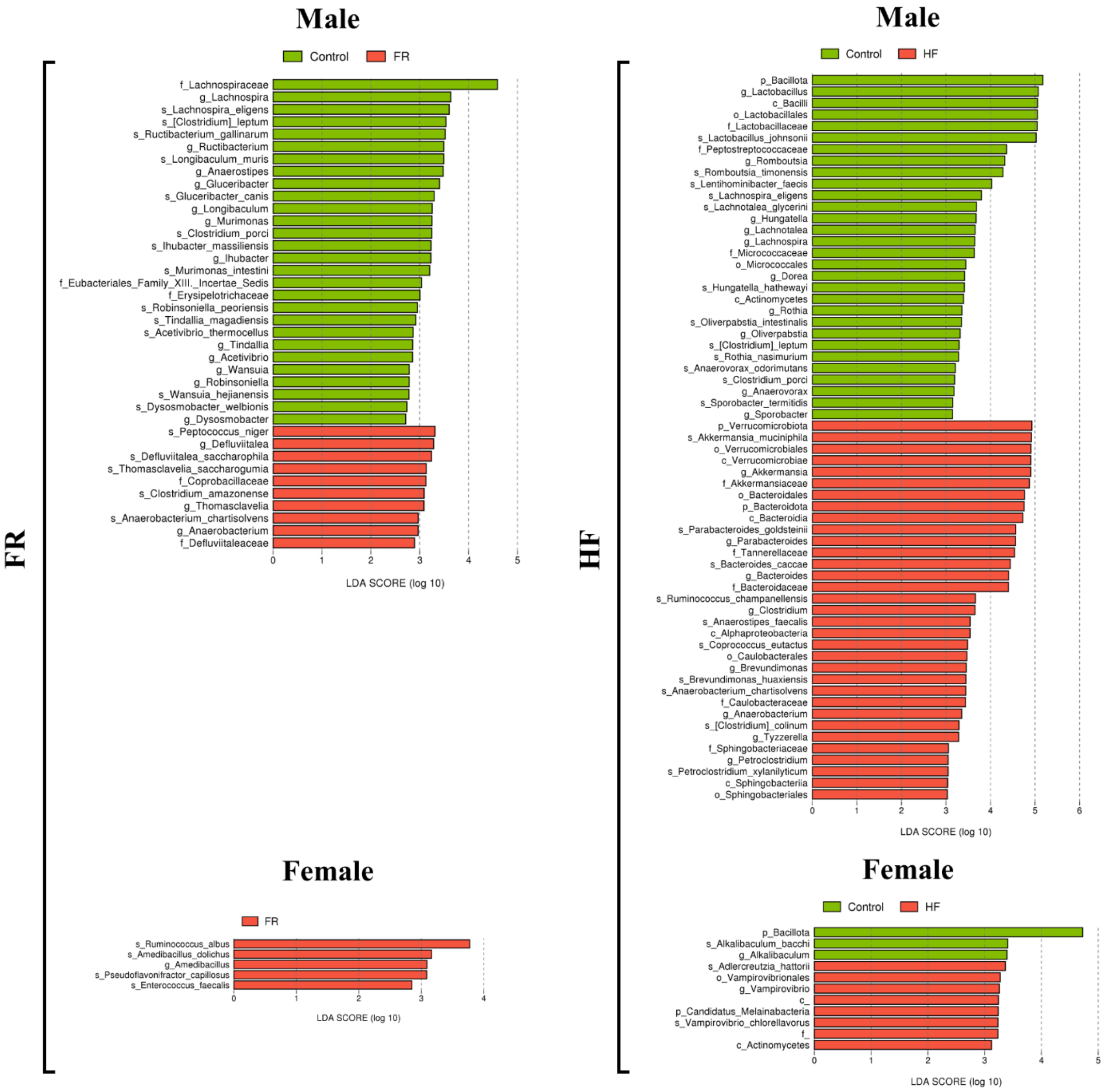

Figure S4. (*Cont.*)

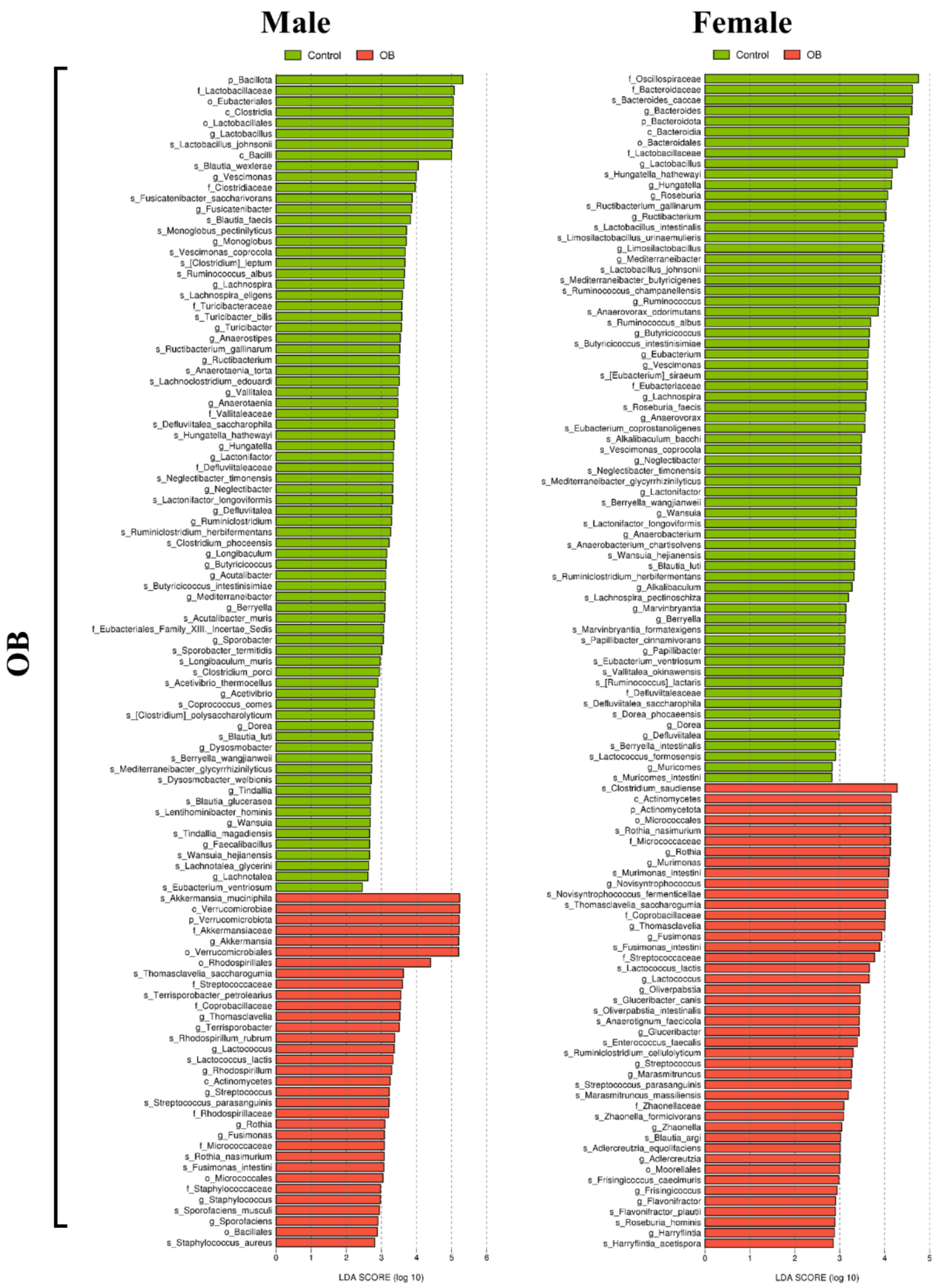

Figure S4. Taxonomy histogram generated by linear discriminant analysis effect size (LEfSe) analysis.

Distinct bacterial taxa in the FR, HF, and OB groups compared to controls, as identified by LEfSe. Histogram displaying the scores of linear discriminant analysis (LDA > 2).

Table S1. Composition of the standard and 45% high-fat diets

Content	Standard diet<br>Kcal (%)	45% high-fat diet<br>Kcal (%)
Carbohydrate	59	35
Protein	27	20
Fat	14	45
**total**	**100**	**100**
